# Comparative Analysis of the Glutathione S-Transferase Gene Family of Four *Triticeae* Species and Transcriptome Analysis of GST Genes in Common Wheat Responding to Salt Stress

**DOI:** 10.1155/2021/6289174

**Published:** 2021-02-18

**Authors:** Yongchao Hao, Shoushen Xu, Zhongfan lyu, Hongwei Wang, Lingrang Kong, Silong Sun

**Affiliations:** State Key Laboratory of Crop Biology, College of Agronomy, Shandong Agricultural University, Taian 271018, China

## Abstract

Glutathione S-transferases (GSTs) are ancient proteins encoded by a large gene family in plants, which play multiple roles in plant growth and development. However, there has been little study on the GST genes of common wheat (*Triticum aestivum*) and its relatives (*Triticum durum*, *Triticum urartu*, and *Aegilops tauschii*), which are four important species of *Triticeae*. Here, a genome-wide comprehensive analysis of this gene family was performed on the genomes of common wheat and its relatives. A total of 346 GST genes in *T. aestivum*, 226 in *T. durum*, 104 in *T. urartu*, and 105 in *Ae. tauschii* were identified, and all members were divided into ten classes. Transcriptome analysis was used to identify GST genes that respond to salt stress in common wheat, which revealed that the reaction of GST genes is not sensitive to low and moderate salt concentrations but is sensitive to severe concentrations of the stressor, and the GST genes related to salt stress mainly come from the Tau and Phi classes. Six GST genes which respond to different salt concentrations were selected and validated by a qRT-PCR assay. These findings will not only provide helpful information about the function of GST genes in *Triticeae* species but also offer insights for the future application of salt stress resistance breeding in common wheat.

## 1. Introduction

Glutathione S-transferases (GSTs; E.C. 2.5.1.18) are ubiquitous enzymes that form a large gene family with a range of functions in plant growth and development. The associated superfamily of proteins can be divided into ten classes [1–3], nine of which are soluble, including Tau, Phi, Lambda, dehydroascorbate reductase (DHAR), Theta, Zeta, *γ*-subunit of translation elongation factor 1B (EF1B*γ*), tetrachlorohydroquinone dehalogenase (TCHQD; [3]), and OMEGA [4]. The exceptions are the microsomal proteins (membrane-associated proteins in eicosanoid and glutathione metabolism, MAPEG). Although the sequences of all soluble GST proteins differ significantly between these classes, their folded structures are similar [5]. Among the ten classes, Tau and Phi GSTs are plant-specific and the most abundant [6]; they play vital roles in metabolizing xenobiotics. Zeta and Theta class GSTs have been highly conserved over a long evolutionary period and have very restricted activities toward xenobiotics [7, 8]. Both DHAR and Lambda class GSTs function as thiol transferases, by replacing their serine residue with cysteine [1, 9, 10]. EF1B*γ* and TCHQD class GSTs account for a very small percentage of the GSTs identified; they have been found in *Brachypodium distachyon*, *Gossypium raimondii*, and *Gossypium arboretum* [11, 12], and the number varied from 1 to 2. The functions of OMEGA class GSTs are very similar to those of the Zeta and Theta classes, which exhibit thiol transferase activity and catalyze the reduction of S-phenacylglutathiones, dehydroascorbate, and methylated arsenic species [13]. MAPEG 50 class GST function is focused mainly on xenobiotic detoxification and antioxidant defense [14]. GSTs were initially discovered because of their ability to detoxify herbicides [15], and their functions can be demonstrated using the 1-chlorine-2,4-dinitronybenzene (CDNB) assay, where CDNB chlorine is replaced by glutathione [16, 17].

In addition to environmentally noxious agents such as herbicides, reactive oxygen species (ROS), which are produced by different abiotic stresses, pose a threat to plant growth and survival [18, 19]. GSTs are important in detoxifying hazardous chemicals and reducing ROS-based stress by catalyzing the conjugation of the tripeptide glutathione to a variety of electrophilic, hydrophobic, and xenobiotic compounds to form a polar S-glutathionylated reaction product [16, 20]. The conjugated reaction product can then either be sequestered in vacuoles or exported from the cell through putative ATP-dependent pump systems [6].

Salt stress is a severe abiotic stress that causes a great deal of damage to the growth and development of crops [21]. In addition to the increased production of ROS, salinity imposes nutritional imbalances, as well as both ionic and osmotic stresses, on tissues, which lead to enormous crop production losses [22]. Identifying genes related to salt resistance and applying them to crop breeding is an effective way to solve the problem of salt stress. GSTs are critical to the acclimation of plants such as *Arabidopsis thaliana* to salt stress [23]. For example, the overexpression of *GsGST* from *Glycine soja* enhances drought and salt tolerance in transgenic tobacco [24], and the expression of GmGSTL1 from soybean in transgenic *A. thaliana* alleviates the symptoms of salt stress [25].

Common wheat is a fundamental and important cereal that provides about 20% of dietary protein and calories worldwide [26] and is cultivated more than any other crop, with a high annual production of 722.4 million metric tons [27]. The common wheat (*Triticum aestivum*) genome is comprised of three homologous and highly similar subgenomes (AABBDD; 2*n* = 6*x* = 42). According to widely accepted findings, common wheat originated from two natural hybridization events [28, 29]. Firstly, a tetraploidization from the hybridization between wild *T. urartu* (AA; 2*n* = 2*x* = 14) and an unknown close relative of *Aegilops speltoides* (BB; 2*n* = 2*x* = 14) resulted in the tetraploid wild emmer wheat (*T. turgidum ssp. dicoccoides*; AABB; 2*n* = 4*x* = 28); then, this species hybridized with *Ae. tauschii* (DD; 2*n* = 2*x* = 14) to form modern hexaploid common wheat. The genome of durum wheat (DW; *T. turgidum L. ssp. durum*; AABB; 2*n* = 4*x* = 28) consists of two closely associated subgenomes, which evolved from domesticated emmer wheat (*T. turgidum ssp. dicoccum*) and wild emmer wheat [30, 31]. Wheat and its relatives are ideal models for plant polyploidy research, and with the release of the genome sequence for hexaploid *T. aestivum* [32], tetraploid *T. durum* [33], and the two diploid species *T. urartu* [34] and *Ae. tauschii* [35], the genome-wide analysis of all related genes in wheat and its relatives can be realized.

In order to analyze the GST genes in common wheat and its relatives, we comprehensively identified and characterized the GST genes in four *Triticeae* species. We also exhibited the syntenic correlation between wheat ABD subgenomes, which will help to better the understanding of the polyploidization process in this gene family. Further, we investigated the function and expression patterns of common wheat GST genes in response to salt stress, which will provide helpful information in the breeding of common wheat for salt stress resistance in the future.

## 2. Materials and Methods

### 2.1. Sequence Search and Identification of GST Genes

The genome sequences and gene annotations of common wheat (*T. aestivum*) were downloaded from website https://wheat-urgi.versailles.inra.fr/Seq-Repository/Annotations, durum wheat (*T. durum*) from https://www.interomics.eu/durum-wheat-genome, *Ae. tauschii* from http://aegilops.wheat.ucdavis.edu/ATGSP/annotation/, and *T. urartu* from MBKBASE website (http://www.mbkbase.org/Tu/). The GST protein sequences of *A. thaliana* (61 numbers) and *Oryza sativa* (80 numbers) were downloaded from The Arabidopsis Information Resource (TAIR, http://www.arabidopsis.org) and the Rice Genome Annotation Project Database (RGAP, http://rice.plantbiology.msu.edu/index.shtml), respectively (Table S1). These sequences were then used as queries in a BLASTP search with the *E* value cutoff of 1*e* − 20 against the gene protein sequences of common wheat and its relatives. Afterwards, nonredundant significant hits in wheat and its relatives were submitted to the Pfam database (https://pfam.xfam.org/) to confirm the presence of the conserved domains. The NCBI conserved domain database (CDD, https://www.ncbi.nlm.nih.gov/cdd) and InterPro database (http://www.ebi.ac.uk/interpro/) were applied to further confirm the candidate genes.

### 2.2. Phylogeny, Chromosomal Distribution, and Synteny Analysis

Multiple sequence alignments of all the identified GST protein sequences were performed using the MUSCLE [36] program with default parameters. Phylogenetic trees were constructed using MEGA X software with the neighbor joining method [37] and the following parameters: bootstrap (1000 replicates) and Poisson model.

All the identified GST genes in wheat and its relatives were located on the pseudochromosomes based on the physical location information acquired from the genome database. To understand the relationship between the GST genes identified in wheat and its relatives at the genomic level, the common and durum wheat genomes were split into three and two diploid subgenomes, respectively. Collinear analysis was then carried out using the five subgenomes with diploid *T. urartu* and *Ae. tauschii* genomes using JCVI software (https://github.com/tanghaibao/jcvi/wiki). To explore the orthologous relationships within the common wheat genome, three subgenomes of wheat were analyzed in the same way, and the results were visualized by Circos [38].

### 2.3. Salt-Treated Transcriptome Library Construction

To investigate the expression patterns of GST genes in wheat under salt stress, a common wheat cultivar (Chinese Spring) was planted in a growth chamber at 25°C under a photoperiod of 16 h/8 h (light/dark). The seedlings were subjected to salt treatment at concentrations of 0, 100, 200, and 300 mM NaCl at the one-week stage. The leaf tissues were harvested after one week of treatment and stored at -80°C, after freezing in liquid nitrogen. The total RNA of all the collected samples was extracted using an RNAprep Pure Plant Kit (TIANGEN, Beijing, China). A NanoDrop 1000 spectrophotometer was used to determine the quantity and quality of the RNA. A total of 12 wheat samples (three biological replicates were conducted for each treatment) were sequenced at Novogene Co. Ltd. (Beijing, China), and paired-end sequencing was performed with an Illumina HiSeq™ 2500 platform (Illumina, USA). After filtering low-quality reads and adaptors, a total of 69.5 Gb of clean data were obtained.

### 2.4. Differential Expression Analysis and qRT-PCR

The RNA-seq reads were first aligned to the reference genome of wheat (IWGSC v1.1) by HISAT2 [39]. HTseq-count [40] was then used to calculate the read count in each sample, and differential expression genes were identified by DESeq2 [41]. The abundance of transcripts was calculated by a custom perl script. Genes with more than twofold differential expression (∣Log2FoldChange | >1) and *P* value < 0.05 were classified as significant differential expression genes (for convenience, significant DEGs are abbreviated as DEGs in this paper). A LightCycler 96 system (Roche, Mannheim, Germany) was used for the qRT-PCR assay with SYBR qPCR Master Mix (Vazyme, Nanjing, China); three technical replicates were carried out. Primers (Table S2) for qRT-PCR analysis were designed with Primer-BLAST (https://www.ncbi.nlm.nih.gov/tools/primer-blast/) based on coding sequences from wheat reference genome annotations (IWGSC v1.1).

## 3. Results

### 3.1. GST Genes Belong to Well-Defined Subfamilies

**Figure 1:**
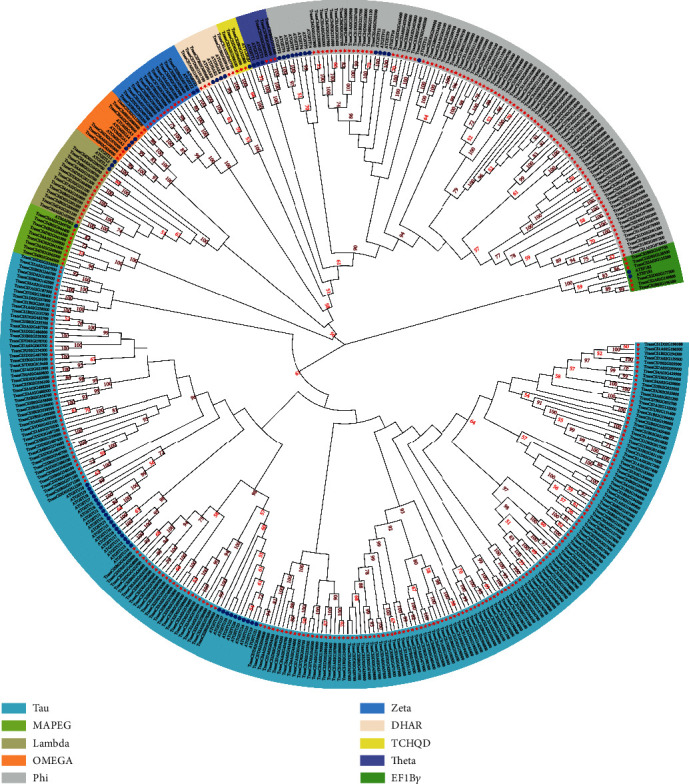
Phylogenetic analysis of glutathione S-transferase genes in *T. aestivum* and *A. thaliana*. The phylogenetic tree was constructed using the neighbor joining method by MEGA-X software; bootstrap scores of >50% are displayed; *T. aestivum* and *A. thaliana* genes are represented by a red star and blue circle, respectively. Different background colors represent different classes.

To study the phylogenetic relationships of the GST family, an unrooted phylogenetic tree was constructed using the GST protein sequences identified in wheat and GSTs predicted in *A. thaliana* (Figure 1, Table S1). All GSTs in each species were divided into the following ten classes: Tau, Phi, Theta, Zeta, Lambda, EF1B*γ*, DHAR, TCHQD, OMEGA, and MAPEG (Figure 1, Table S3). The GST genes that belong to the same classes clustered very well with those from *A. thaliana* (Figure 1). The Tau class GSTs accounted for the majority, followed by the Phi class, which is consistent with findings in other plants such as soybean [42], rice [43], and pepper [44]. The automatic annotation of the genome often produces many errors. We manually checked the GST gene sequences of wheat and its relatives and summarized the wrongly annotated genes based on the homology relationship between the four species (Table S3).

### 3.2. Features of GST Subfamilies in Common Wheat and Its Relatives

**Figure 2:**
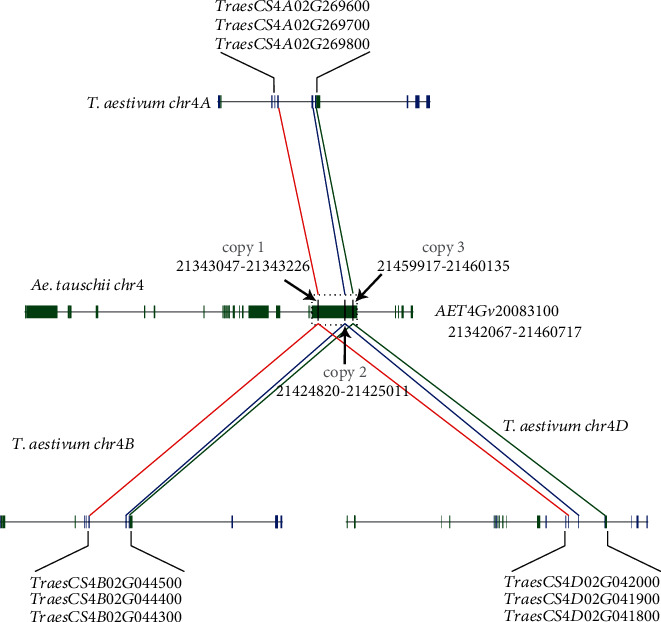
Collinear analysis of MAPEG genes in *T. aestivum* and *Ae. tauschii*. Orthologous MAPEG class genes are linked by lines of the corresponding color (red, blue, and green). Three high similarity regions of the *AET4Gv20083100* gene are surrounded by a black dashed box.

We analyzed the number of each class of GST genes in common wheat and its relatives (Table 1). Brought together, the number of GST genes is directly related to the ploidy of their genomes. However, the gene number of the MAPEG class between *T. aestivum* and *Ae. tauschii* was found to be abnormal. The MAPEG class was expected to have three copies in the *Ae. tauschii* genome, but only one gene was identified. Therefore, we conducted a microsynteny analysis between the three subgenomes of common wheat and *Ae. tauschii* (Figure 2), which showed that the MAPEG genes identified in common wheat had good collinearity with the three genomic regions associated with the MAPEG gene (*AET4Gv20083100*) in *Ae. tauschii*. Furthermore, the MAPEG gene (*AT1G65820*) in *A. thaliana* was used as a query in a TBLASTN search (*E* value < 1*e* − 20) against *Ae. tauschii* genome sequences. The result showed that *AT1G65820* has high similarity with three genomic regions of *AET4Gv20083100* (chr4D: 21,343,047–21,343,226, 21,424,820–21,425,011, and 21,459,917–21,460,135; Table S4). According to the genome annotation file, *AET4Gv20083100* is a very long gene (118.65 kb, chr4D: 21,342,067–21,460,717), with 14 transcripts, which greatly exceeds the size of normal genes. We therefore confirmed that this is a genome annotation error, and there are actually three copies of MAPEG genes in this genomic interval.

### 3.3. Chromosomal Distribution and Synteny Analysis

**Figure 3:**
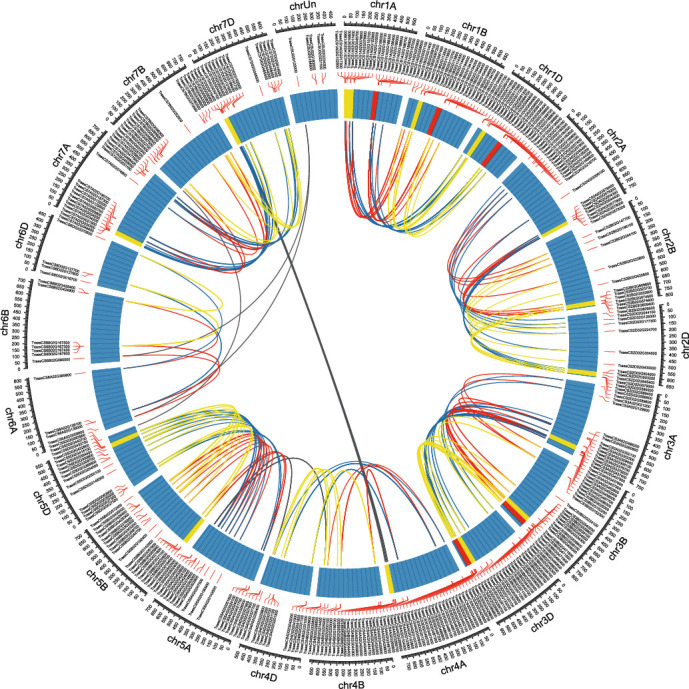
Genome-wide synteny analysis of glutathione S-transferase (GST) genes in common wheat. Syntenic GST gene pairs belonging to the same linkage group between AA and BB, AA and DD, and BB and DD are linked with red, blue, and yellow lines, respectively. Syntenic GST gene pairs between different linkage groups are linked with gray lines. The heat map track indicates the density distribution of GST genes.

**Figure 4:**
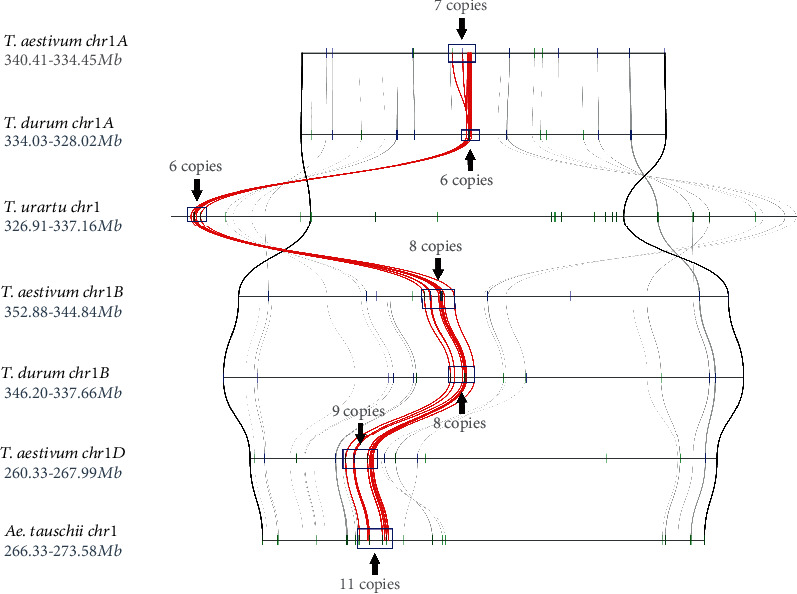
Microsynteny analysis of a glutathione S-transferase (GST) gene cluster on chromosome 1 of common wheat and its relatives. Syntenic GST genes are linked by red lines; the chosen genomic border is represented by black lines, and others by gray lines.

Genome-wide synteny analysis exhibited a high level of collinearity between the GST genes in the three subgenomes of wheat, and the GST genes were seen to be distributed mainly in the distal region of each chromosome (Figure 3). A microsynteny analysis of a GST gene cluster on chromosome 1 further showed that common wheat and its relatives have good synteny, with unequal gene numbers (Figure 4). Subgenome A of common wheat has one more copy than *T. urartu* and subgenome A of *T. durum*; subgenome B of common wheat has the same number of GST genes as subgenome B of *T. durum*; and *Ae. tauschii* has two more copies than subgenome D of common wheat. Indeed, because of the close relationship between the four species, the phenomenon of unequal gene numbers is more likely due to the genome annotation being not perfect. However, *T. urartu*'s GST gene cluster is located outside this genomic region (Figure 4); this may be an inversion in the *T. urartu* genome. It also may be an assembly error in the *T. urartu* chromosome, since *T. urartu* is ancestral to the *T. aestivum* and *T. durum* and the inversions are not apparent on the A chromosomes from *T. aestivum* and *T. durum*, and not on the homeologous B and D genome chromosomes.

### 3.4. Expression Analysis of the GST Genes under Salinity Treatment

**Figure 5:**
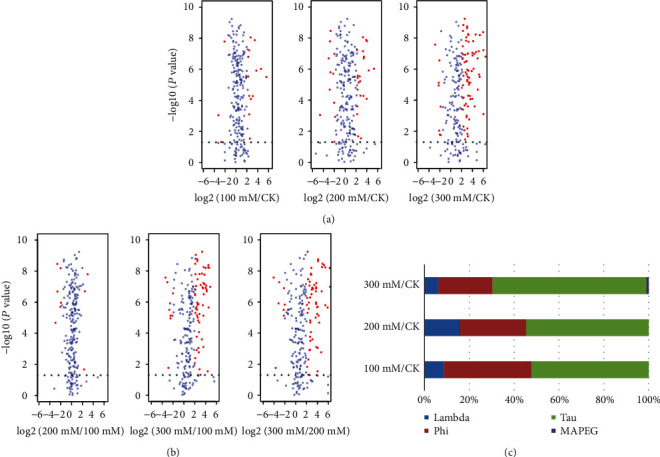
Expression analysis of all DEGs in various salt concentrations. (a, b) Differentially expressed glutathione S-transferase genes under salt stress in common wheat. (c) The proportion of DEGs in different classes. Red dots represent DEGs, and blue dots represent non-DEGs in (a, b). Different classes are represented by corresponding colors in (c).

Salt is an extremely threatening environmental stress for most plants, but little is known about the response of GST genes to salt stress in common wheat. Therefore, we analyzed the expression patterns of GST genes in common wheat, using transcriptome data. A total of 320 GST genes were expressed under salt stress, and these were used for further analysis (Table S5). The number of DEGs showed significant differences compared to the control sample under slight stress (100 mM NaCl), moderate stress (200 mM NaCl), and severe stress (300 mM NaCl; Figure 5(a)). Under 100 mM NaCl, 22 GST genes were upregulated and four were downregulated; under 200 mM NaCl, 50 were upregulated and 17 were downregulated; and at 300 mM NaCl, there was a significant increase in the number of DEGs. At this point, of the 112 DEGs, 103 were upregulated and 15 were downregulated (Table S6). We then compared the expression changes of GST genes under different treatments (200 vs. 100 mM NaCl, 300 vs. 100 mM NaCl, and 300 vs. 200 mM NaCl; Figure 5(b)). 21 GST genes were upregulated and 13 were downregulated in plants exposed to 200 mM salt compared with those exposed to the lowest concentration, suggesting that there was no significant change in the number of DEGs at these two concentrations. In the comparisons of the other two groups (300 vs. 100 mM NaCl and 300 vs. 200 mM NaCl), 109 and 111 DEGs were identified, of which 90 were upregulated in the two compared groups, whereas 8 and 9 were downregulated, respectively (Table S7). Moreover, based on the FPKM value of all the expressed GST genes (Table S8), low-expression genes accounted for the majority at the low and moderate salt concentrations, but at 300 mM salt, most GST genes had high expression values. The results indicated that the reaction of GST genes is not sensitive to low and moderate salt concentrations but is sensitive to high concentrations of the stressor.

Among the 23 DEGs identified in plants exposed to a low concentration of salt, 12 belong to the Tau, nine to the Phi class of GSTs, and two to Lambda class. And for the 64 DEGs that were exhibited at the moderate salt concentration, 35 belong to the Tau, 10 to the Lambda, and 19 to the Phi class of GSTs. Of the 112 DEGs identified in plants exposed to a severe concentration of salt, one belongs to the MAPEG class, whereas seven belong to the Lambda, 27 to the Phi, and 77 to the Tau class of GSTs (Figure 5(c) and Table S9). In summary, the DEGs were mainly from the Tau and Phi classes, which is consistent with the result of previous reports [45] in other plants. In summary, GST genes related to abiotic stress mainly come from the Tau and Phi classes.

### 3.5. Validation of DEGs by qRT-PCR

**Figure 6:**
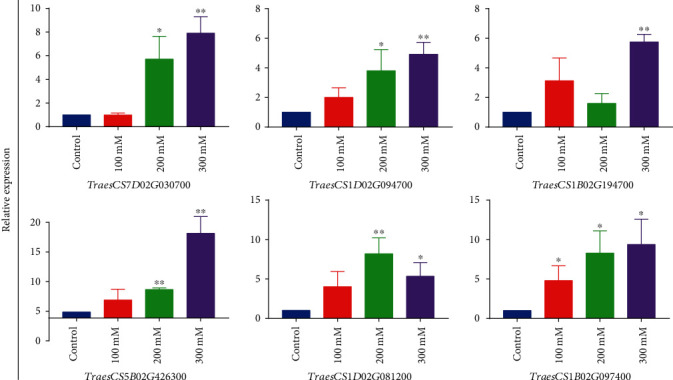
Relative expression of the selected glutathione S-transferase genes, as analyzed by quantitative real-time PCR. Different salt concentrations are represented by corresponding colors (blue (control), red (100 mM), green (200 mM), and purple (300 mM)). The error bars represent the standard deviation (S.D.) of the means of three independent replicates. Compared to the control group, statistically significant differences referenced to ^∗^*P* < 0.05 and ^∗∗^*P* < 0.01 by Student's *t*-test.

To verify the results of the transcriptome analysis, six DEGs that have a high expression level under different salt concentrations (Traescs7D02G030700, TraesCS1D02G094700, TraesCS1B02G194700, TraesCS5B02G426300, TraesCS1D02G081200, and TraesCS1B02G097400) were selected for quantitative real-time (qRT) PCR assay analysis (Figure 6). The results showed that the expression levels of TraesCS1D02G094700, TraesCS5B02G426300, and TraesCS1B02G097400 increased along a significant gradient with the three salt concentrations. The expression of TraesCS1B02G194700 was inhibited at 200 mM but increased at 100 and 300 mM. With regard to Traescs7D02G030700, the expression level did not change significantly at 100 mM but significantly increased at 200 and 300 mM. As for TraesCS1D02G081200, the expression level showed a tendency to increase at first and then decrease with increasing salt concentrations. Overall, the RT-PCR results agree with the RNA seq data, and these results indicated that there are many different patterns of GST gene expression in response to salt stress in common wheat; the diversity in the expression patterns is related to the diversity in gene functions. And combined with the result of RNA-seq analysis, with the change of salt concentration, the types and number of GST genes responding to salt stress also changed, which to some extent reflects the adaptability of the GST gene family of common wheat to salt stress.

## 4. Discussion

Many GST genes have been reported to play central roles in protecting plants from abiotic and biotic stress [19, 46–48]; therefore, they are potential targets for crop breeding and improvement.

In this study, we identified a total of 346 GST genes in hexaploid common wheat (*T. aestivum*), 226 in tetraploid durum wheat (*T. durum*), 104 in diploid *T. urartu*, and 105 in diploid *Ae. tauschii*, using a comprehensive genome-wide approach. The numbers of GST genes identified in these four species basically conform to their genome ploidy, which indicates that the GST gene family was conserved during the process of polyploidization. In a recent study, the GST gene family in common wheat was divided into eight classes [49], but there is evidence that the GST gene family also includes the OMEGA and MAPEG classes in plants [1, 50, 51]. To accurately identify the GST genes in common wheat and its relatives, we updated the classification of the GST genes and largely expanded the membership of this gene family.

Salt stress is severe abiotic stress and does great damage to crops; therefore, many studies focus on the ability of GSTs to resist salt stress in plants. Some salt stress resistance GST genes have previously been identified in plants [24, 52, 53], which offered guidance for the mining of salt resistance GST genes in wheat. So, we designed experiments based on salt stress in common wheat and identified DEGs associated with salt resistance through a rigorous transcriptome analysis process. Based on the differential expression analysis, we determined that the number of DEGs, especially the number of upregulated genes, rises significantly under a severe concentration of salt. Six DEGs which respond to different salt concentrations were identified and further confirmed by qRT-PCR assay data. Although they have been identified by bioinformatics methods, the evidence is not sufficient. How and to what extent these two genes play a role in wheat still needs to be verified by designing biological experiments. Overall, the information provided by this study will provide a basis for further assessment of the biological roles of GST genes in common wheat and also may be useful to wheat breeding programs in the future.

Gene duplication plays an important role in gene family expansion in plants [51, 52]. In general, GST genes expanded mainly by tandem duplication in all the analyzed species, which indicates that this is the main driving force for GST gene family expansion in wheat and its relatives. Distal telomeric segments in the chromosome were described as targets of recombination events, and many fast-evolving genes lie within these evolutionary hotspots [54, 55]. Genes in wheat specifically related to stress response and external stimuli, notably traits with a high requirement for adaptability, have previously been found to be located in distal telomeric segments [56]. And genes related to cell cycle, photosynthesis, or translation are enriched in proximal chromosomal segments. As these four closest species have similar characteristics in their genome. This finding is supported in this research: GST genes tend to be located in distal chromosome segments, and the GST gene family in wheat and its relatives expands mainly by tandem duplication. Hence, we inferred that the main reason for the presence of GST gene clusters may be the preference of plants to retain these genes during evolution and the higher prevalence of duplication events possibly facilitating rapid adaptation to different environmental conditions.

## Figures and Tables

**Table 1:** Number of glutathione S-transferase genes identified in wheat and its relatives.

Class	*T. aestivum*	*T. durum*	*T. urartu*	*Ae. tauschii*
Tau	200	133	61	62
Phi	87	56	25	28
Lambda	14	8	4	3
Zeta	13	9	3	3
Theta	3	1	1	1
DHAR	5	3	2	2
EF1B*γ*	6	4	2	2
TCHQD	3	2	1	1
OMEGA	6	4	2	2
MAPEG	9	6	3	1 (3)
Total	346	226	104	105

## Data Availability

The transcriptome data used in this study have been uploaded to NCBI under BioProjectID PRJNA632706. The other data used to support the findings of this study are available from the corresponding author upon request.

## Supplementary Materials

Supplementary Materials Table S1: the GST genes of *Arabidopsis thaliana* and *Oryza sativa*. Table S2: primers used in this study. Table S3: the GST genes identified in wheat and its relatives. Table S4: the results of a BLASTN search against the *Ae. tauschii* genome sequence using the MAPEG gene (*AT1G65820*) from *A. thaliana* as a query. Table S5: differentially expressed GST genes at different salt concentrations. Table S6: identification of differentially expressed genes between various salt concentration treatments and the control. Table S7: identification of differentially expressed genes between various salt concentration treatments. Table S8: FPKM value of all GST genes in common wheat. Table S9: classification of DEGs under various salt concentrations.
